# “They Don't Understand People With Learning Disabilities”: Exploring the Experiences of People With Intellectual Disabilities Undergoing Welfare Assessments

**DOI:** 10.1111/jar.70000

**Published:** 2025-01-07

**Authors:** Bethan Ward, Ste Weatherhead, Beth Greenhill

**Affiliations:** ^1^ Department of Primary Care and Mental Health Liverpool UK; ^2^ Mersey Care NHS Foundation Trust Prescot UK

**Keywords:** benefits‐distress, people with intellectual disabilities, qualitative, stigma, welfare assessment

## Abstract

**Background:**

The Welfare Reform Act (2012) has been criticised for harming claimants, particularly through functional assessments. Although many people with intellectual disabilities in the UK receive welfare benefits, their experiences of undergoing functional assessments are under‐researched.

**Method:**

Eight participants with intellectual disabilities were interviewed about experiences of welfare assessment. Transcripts were analysed qualitatively using interpretative phenomenological analysis.

**Results:**

Analysis suggested five group experiential themes: ‘Living in fear: I was nervous and scared’; ‘The system is marginalising: Other people are better than me’; ‘Relationship with the assessor: His attitude fucking stunk’; ‘Others as a safe base: Someone there that you know, and you trust’; and ‘Responding with empowerment: That's where I really shined’.

**Conclusions:**

People with intellectual disabilities experience functional assessments as scary and oppressive. Assessment reinforced the stigma associated with having an intellectual disability and, to a lesser extent, claiming benefits. Individual, structural and policy levels interventions are discussed.

## 1 Introduction

Around 95% of adults in the UK with an intellectual disability are unemployed (Office for National Statistics 2019; Hatton 2019; NHS England 2023). Those who are unemployed often rely on financial support from state welfare benefits.

The initial aims of the UK welfare system vary significantly from its current operationalisation. The post‐WW2 welfare state (Beveridge 1942) aimed to tackle unemployment and sickness following the 1930's economic depression and Second World War (Deeming 2019), and was designed to meet the needs of the poorest people (Titmuss 1974). From the financial crises of the 1970's to the ‘austerity’ driven by the banking crisis of the early 2000's, changes in the ideological approach to welfare have accompanied reduced welfare spending (Greve 2022). While welfare was intended to support the poorest and sickest, the current neoliberal political agenda often seeks to justify removing the welfare state, by increasing conditionality (Thornton and Iacoella 2024) and stigmatising people claiming benefits (Baumberg 2016; Garthwaite 2015).

The impact of claiming welfare benefits is an emerging area of interest following the most recent UK Welfare Reform Act (2012), which included increased conditions for benefits to be received (DWP 2010). Increased conditionality has been criticised for causing harm to claimants (Arie 2018), creating psychological distress (Wickham et al. 2020) and exacerbating mental health conditions (Cheetham, Moffatt, and Addison 2018). Moth, Hart, and Greener (2020) view this as a punitive welfare approach and a form of social harm.

A key change was the introduction of functional assessments to assess benefit eligibility (Harris 2024). The work capability assessment (WCA) is utilised for Employment Support Allowance (ESA) and Universal Credit (UC), assessing an claimant's ability to work, while the Personal Independence (PIP) assessment focuses on the impact of a health condition upon an individual's care or mobility needs (DWP 2010, 2016).

WCA have been associated with increased suicides, mental health symptoms and antidepressant prescribing (Barr et al. 2016). Claimants reported exacerbation of existing health issues (Dwyer et al. 2016) and deterioration in mental health, including thoughts of suicide (Marks, Cowan, and McLean 2017). Distress has been linked to completing forms, errors in reports, lack of assessor expertise (House of Commons 2018), communication with benefit officials, medical assessment and the claims process (Machin and McCormack 2023). Pybus et al. (2021) found that in claimants who access support for their mental health, functional assessments were experienced as stressful and sometimes led to suicidal thoughts, which was linked to fear, insecurity and disempowerment.

Cheetham, Moffatt, and Addison (2018) suggested potential psychological mechanisms underlying links between welfare reform and distress, including the negative impact on self‐esteem, evoking feelings of helplessness and despair. Moth and Lavalette (2017) proposed a range of psychological mechanisms underlying benefits‐distress in their sample of people with mental health difficulties undergoing WCA (Table 1).

**TABLE 1:** Mechanisms and impacts underlying mental health difficulties related to claiming benefits (Moth and Lavalette 2017).

Mechanism	Description
Re‐traumatisation	Disclosing intensely personal and sensitive experiences was as highly distressing. Some participants felt forced to disclose and relive traumatic experiences and were not offered specialist or therapeutic support
Invalidation	Participants felt their experiences were disregarded and undermined in the assessment. The credibility of their experiences was questioned
Fear	Participants described fear triggered by invitation to assessment and fear of sanctions (e.g., benefits being stopped if not meeting eligibility conditions)
Mistrust	Participants felt that the assessment process was designed to trip them up and trap them through misrepresentation of their experiences, which led to not trusting the system
Double Binds	A type of ‘trap’ experienced by participants characterised by contradictory injunctions and conflicting messages (e.g., requested to travel for WCA but presentation at location cited as invalidation for their claim to be incapacitated due to ability to travel)
Shaming and Blaming	Participants described experiences of shame directly linked to welfare policy. They felt they were stigmatised as useless compared to working people

People with disabilities may be disproportionately impacted by welfare reform (McGrath et al. 2016; Garthwaite 2014), with higher levels of reported uncertainty and anxiety (Baumberg 2016), possibly linked to feelings of powerlessness around assessment outcomes (De Wolfe 2012; Ploetner et al. 2020). PIP replaced Disability Living Allowance (2012) and included functional assessments conducted by private companies (DWP 2016). Multiple inquiries have raised concerns after finding PIP claimants did not feel listened to or understood during the assessment (Gray 2014, 2017; United Nations 2016). The British Psychological Society (BPS 2016) suggested the PIP assessment failed to recognise the highly individualised nature of people's experiences, reflected in claimant's experiences of PIP assessments as dehumanising, difficult to understand, and complex (Allen et al. 2016). Furthermore, disabled claimants report experiences of humiliation and shame in relation to claiming welfare (Garthwaite 2014; Ploetner et al. 2020).

Redley (2009) and Baumberg et al. (2015) argued that benefit assessments ignore restricted employment opportunities for those with intellectual disabilities, failing to address the lack of services that support self‐development. People with intellectual disabilities are at risk of being socially excluded, unable to fulfil the responsibility of employment constituting mainstream citizenship within neoliberal politics (Hall 2010; Goodley 2016). Although people with intellectual disabilities both want and benefit from inclusive employment, the assumptions of repeated functional assessments seem incompatible with the notion of lifelong disability. Furthermore, Litchfield (2014) reviewed WCA in the context of claimants with intellectual disabilities and suggested that there may be a propensity to overstate or seek to please interviewers, which could lead to misrepresentation of a claimant's needs.

Evidence suggests claiming benefits is stigmatising (Baumberg 2016; Garthwaite 2015; Saffer, Nolte, and Duffy 2018; Pemberton et al. 2016). Stigma refers to widely endorsed negative stereotypes held by those with social power, resulting in discrimination and loss of status for specific groups (Link and Phelan 2001). Benefit stigma has been linked to negative impacts on self‐esteem (Pemberton et al. 2016), shame, social withdrawal (Garthwaite 2015), and psychological distress (Patrick 2016).

Some researchers argue unemployment has been positioned as an individual and personal failure, rather than a product of socio‐economic factors, to better serve current dominant neoliberal approaches (Friedli and Stearn 2015). This is maintained by behavioural approaches such as surveillance, deterrence, sanctions, and stigma (Wacquant 2009, as cited in Moth, Hart, and Greener 2020). ‘Weaponising stigma’ (Scambler 2018) describes how this narrative of unemployment as an individual failure has been used as a political tool to justify the limitations of the welfare state to the public.

Research suggests people with intellectual disabilities are already highly stigmatised (Scior and Werner 2016; Redley 2009) and perceived as severely cognitively and socially impaired and childlike (McCaughey and Strohmer 2005; Gilmore, Campbell, and Cuskelly 2003). Roth, Peretz, and Barak (2016) found that people with intellectual disabilities felt ridiculed by others, often not understanding why they are treated differently to others. People with intellectual disabilities' perception of stigma has been linked to low self‐esteem (Paterson, McKenzie, and Lindsay 2012) and psychological distress (Ali et al. 2015; Dagnan and Waring 2004).

Low rates of paid employment for people with intellectual disabilities in the UK (4.8%; NHS England 2023) may be in part due to stigma. Shaw et al. (2004) found evidence that the public believe people with intellectual disabilities to be mostly unemployable. Unemployment may also perpetuate stigma, due to current neoliberal ideology emphasising an individual's responsibility to work to gain citizenship and eligibility for rights (Redley 2009; Goodley 2016). Evidence suggests that stigmatising disablist hate speech on public online forums has increased in line with increased austerity measures (Burch 2018). In contrast to the social‐role enhancing interventions advocated by social role valorisation (Osburn 2006), these factors serve to exclude people with intellectual disabilities from employment and increase reliance on welfare benefits.

Emerging evidence suggests welfare reform (such as the introduction of functional assessments) impacts negatively on claimants. However the intersectional experience of having an intellectual disability and claiming benefits remains to be explored. The current study explored how people with intellectual disabilities make sense of their experience of welfare assessments.

## 2 Method

The current research was submitted as part of the primary researcher's Doctorate of Clinical Psychology thesis (Ward, Greenhill, and Weatherhead 2021) and includes all of the thesis data and findings. The research team consisted of one trainee Clinical Psychologist and two qualified Clinical Psychologists; one with expertise in the field of intellectual disabilities and the other with expertise working with those in receipt of welfare benefits.

### 2.1 Design

Semi‐structured interviews were conducted remotely. Transcribed data were analysed using interpretative phenomenological analysis (IPA; Smith 1996; Smith and Osborn 2008; Smith et al. 2009).

### 2.2 IPA

IPA aligns with the researcher's epistemological stance in seeking subjective and unique understanding, rather than objective reality (Flowers, Hart, and Marriott 1999; Smith and Osborn 2008). Rose et al. (2019) concluded that IPA is appropriate for researching the experience of people with an intellectual disability. Furthermore, the research area is novel and complex, for which IPA is considered particularly useful (Smith and Osborn 2008).

### 2.3 Participants

Eight participants chose pseudonyms to maintain anonymity. Participants included four males and four females aged 28–54. Participants lived in the community and identified with the label ‘learning disability’. Table 2 summarises participant demographics.

**TABLE 2:** Summary of participant demographics.

Pseudonym	Conditions	Current welfare benefits	Current employment	Length of interview (min)
AJ	Intellectual disability; autism	PIP	Employed part‐time	45
Bob	ID	PIP, ESA	None	52
Dave	ID	PIP, ESA	None	57
Jonathan	Intellectual disability; autism	PIP	Volunteer	38
Louise	ID	PIP, ESA	Volunteer	47
Miss Moneypenny	ID	PIP, ESA	Employed part‐time	34
Natasha	ID & autism	ESA	Employed part‐time	55
Sue	ID	PIP	None	47

Abbreviations: ESA, Employment and Support Allowance; PIP, Personal Independence Payment.

### 2.4 Procedure

#### 2.4.1 Ethics

Liverpool University ethical approval was granted prior to recruitment and data collection.

#### 2.4.2 Recruitment

Participants were recruited via two independent charities promoting equality for people with intellectual disabilities. Staff distributed information to charity members satisfying inclusion criteria (see Table 3). Eight potential participants attended a pre‐meeting via ‘Zoom’ with the researcher to learn more and all decided to take part.

**TABLE 3:** Summary of inclusion and exclusion criteria.

Inclusion criteria	Exclusion criteria
Self‐reported intellectual disability Aged 18+ Capacity to consent (if concerns flagged by charity staff or researcher) Experience of a welfare eligibility assessment in the last 30 months Communication skills required for participating in an interview English as first language	High distress levels as indicated by self‐report, information from staff or carers or observation of the researcher

#### 2.4.3 Informed Consent

An accessible participant information sheet (PIS) and consent form were developed following guidance (Health Research Authority 2020) and consultation with people with intellectual disabilities. The PIS was shared and read by the researcher during the pre‐meeting. Six participants agreed to participate during the pre‐meeting, while two confirmed participation the following week. Consent was audio‐recorded following review of the consent form at the beginning of each interview.

#### 2.4.4 Interviews

Semi‐structured interviews took place virtually via Zoom, lasting 34–57 min. Participants were interviewed once, and seven people opted to have a staff member present for the interview. Questions were generally open‐ended and exploratory, and the schedule was intended as a guide only. As varying levels of communication were expected within the sample, prompts were devised. Following consultation with people with intellectual disabilities, an easy read interview schedule was offered to participants but none felt they required it.

#### 2.4.5 Reflexivity in IPA

The researcher collected and interpreted data with awareness of their own experiences, pre‐conceptions, and prejudices (Smith et al. 2009; Heidegger 1994; Engward and Goldspink 2020). This contributes to the double hermeneutic of IPA, which refers to the researcher making sense of and interpreting the participant's sense‐making (Smith et al. 2009). This process was supported by a reflexive position statement and a reflective diary. The researcher's epistemological position lies closest to social constructionism, questioning distinct categories such as gender and intellectual disability. Due to this, ‘objective assessments’ of participants' intellectual disability were not used. The lead researcher comes from a working‐class family who received benefits and experienced feeling ‘othered’ by financially stable families. This experience is reflected in the analysis, particularly in the theme of marginalisation. The lead researcher is a systemically‐informed clinical psychologist who is critical of neo‐liberal ideology, which is also reflected in the analysis.

#### 2.4.6 Data Analysis

IPA does not prescribe a single method of data analysis (Smith et al. 2009); the current analysis was informed by Smith and Osborn (2008) and Smith, Flowers, and Larkin (2022). Transcripts were re‐read while listening to the audio recordings before in‐depth line by line coding; descriptive, linguistic and conceptual features were noted, informing individual experiential statements. Qualitative data analysis software ‘NVivo’ was used to store experiential statements and quotes. Experiential statements were re‐organised into relevant personal experiential themes (case by case analysis). Patterns within the full data set were iteratively explored (cross‐case analysis), producing a set of group experiential themes. Analysis was circular and dynamic, exploring different patterns in different ways.

#### 2.4.7 Quality Assurance

An audit trail ensured analysis could be traced to the original data (Yin 1989). The analysis was grounded in the data to ensure credibility (Smith 2011); all themes were evidenced by direct quotes and themes required representation from at least half of the sample (Table 4). Frequent supervisory discussions facilitated ongoing credibility checks (Elliott, Fischer, and Rennie 1999).

**TABLE 4:** Themes across participants.

Themes		Participants							
Group experiential theme	Subtheme	AJ	Sue	Natasha	Louise	Bob	Jonathon	Miss Moneypenny	Dave
Living in Fear	*Assessment*	x	x	x	x	x	x	—	x
	*Financial survival*	x	x	—	x	x	x	—	—
The System Is Marginalising	*Learning Disability Stigma*	x	x	x	x	x	x	x	x
	*Benefit Stigma*	—	—	—	x	—	x	x	x
	*Disempowerment*	x	x	x	x	x	x	x	x
Relationship with Assessor	*Humiliation and Dismissal*	x	x	x	x	x	x	x	x
	*Not Understanding Me*	—	x	x	x	—	—	x	x
Others as Safe Base	*Support from Others*	x	x	x	x	x	x	x	x
	*Degrees of Independence*	—	x	x	x	x	—	x	x
Responding with Empowerment	*Finding Value*	—	x	—	x	x	—	x	—
	*Resistance*	—	x	x	x	—	—	—	x

## 3 Results

Analysis resulted in five closely‐related group experiential themes (GETs): ‘Living in fear,’ ‘The system is marginalising’, ‘Relationship with assessor’, ‘Others as a safe base’ and ‘Responding with empowerment’. Each theme is described below, supported with direct participant quotes. Each GET had several subthemes. Table 4 lists GETs and subthemes across participants. Quotes are rich but quite short, consistent with previous IPAs focussing on people with intellectual disabilities (Rose et al. 2019).

### 3.1 Living in Fear: ‘I Was Nervous and Scared’

A visceral sense of fear was dominant across seven participant's accounts, expressed in two main subthemes.

#### 3.1.1 Fear of the Assessment: ‘I Just Couldn't Wait to Get Out of There’

Seven participants described feeling frightened about the assessment, expressed directly through references to their feelings, physiology and indirectly through their behavioural responses to their emotional states. Participant emotions ranged from acute panic (‘Very scared and panicky’ Jonathan), stress (‘I was almost pulling my hair out at times with the stress’ Bob) and anxiety (‘I was anxious’ AJ).

Jonathan described an intense and embodied experience of anxiety during the assessment; he ‘couldn't sit still’, feeling he had ‘run a marathon’, interpreted as a physical fear response. Sue's language suggested she was angry during the assessment (‘pissed me off’), potentially indicating a ‘fight’ response to fear. Analysis suggested Dave wanted to escape the assessment, interpreted as a ‘flight’ response (‘I just couldn't wait to get out of there’). Louise's response to assessment was overwhelming and uncontrollable:

I do have meltdowns, when I go to [welfare] appointments. (Louise)

The unpredictability of the assessments was interpreted as contributing to fear and anxiety:

I think it just makes me nervous: you don't know what they're gonna ask you, who you're gonna see, how long your appointment's gonna be. (Natasha)

There was also fear about getting the questions wrong:

Like things where you don't know the answer to, don't know how to say, things that make you feel nervous makes and you wanna leave the room. (Jonathan)

Interpretation of participant's language supported this theme. For example, Sue described the assessment as ‘horrible’ throughout her interview; the etymology of the word *horror* is from the Latin word *horrere*, meaning to shudder and tremble in fear. Therefore, Sue's language suggested fear or dread:

That's my experience and… (pause) and horrible experience actually. (Sue)

#### 3.1.2 Fear Related to Financial Survival: ‘How Am I Gonna Live?’

Five participants discussed fear of losing their benefits and of losing their ability to meet their basic human needs:

How am I gonna live? How I'm gonna feed and clothe myself. (Bob)

Jonathan named his biggest fear as becoming homeless, suggesting financial security felt fragile and fleeting:

I always saw like a homeless person on the street and I was just had a massive fear that I don't wanna end up like that.

The risk of the assessment process leading to Jonathan being on the streets feels tangible, terrifying, and outside of his agency or control without any sense of a buffer to protect him from this outcome.

For Bob, the stress of financial insecurity impeded his ability to live his life:

If you're struggling with money and, like I have been, you, you can't really have a life, cos you're stressing about everything else.

Bob's reflections about how ‘your life’ is put on hold in the struggle to survive when poverty permeates every aspect of experience, reflect the pervasive sense of fragility and threat throughout participant's accounts.

### 3.2 The System Is Marginalising: ‘Other People Are Better Than Me’

Analysis suggested that welfare assessments were experienced as marginalising, reinforcing intellectual disability stigma and disempowering participants through removing their sense of agency.

#### 3.2.1 Reinforcing Intellectual Disability Stigma: ‘I Feel Stupid’

Across all participants' accounts, assessment appeared to resonate with a stigmatised view of people with intellectual disabilities as inadequate. Inaccessible DWP correspondence highlighted cognitive difficulties related to the participant's intellectual disabilities:

It's horrible. And especially when you got, get letters from benefits and you don't know what the hell they're talking about. (Sue)

Dave felt hurt to be reminded of his perceived inadequacies; here a shaming, accusatory voice is attributed to the assessor, inviting an imagined other to join in his humiliation when Dave's reading difficulties become apparent:

It was horrible… “oh you can't read or write, look at him”, you know… it hurt me a lot…It does hurt me a lot now.

Miss Moneypenny described a deep sense of shame grounded in comparing herself with others when she was unable to understand DWP letters. Her factual assertion that ‘other people are better than me’ because of their academic achievements may be interpreted to suggest internalised intellectual disability stigma:

It feels horrible, cus really, God, erm, I feel stupid because other people are better than me, because they've gone through school and done better than me, so I feel terrible.

AJ and Bob understood that they had lost their benefits due to performing well and focusing on their strengths in the assessment, a ‘lose‐lose’ situation where rejecting the stigma of inadequacy could lead to loss of welfare:

I still didn't get [benefits] because the woman said I answered every question without difficulty. (Bob)

#### 3.2.2 Reinforcing Benefit Stigma: ‘Pretending to be Disabled’

Four accounts suggested the system reinforced benefit stigma in a variety of ways. Louise described proving her entitlement to benefits during the hostile questioning of the assessment:

This is why I've come, because I've got learning disabilities, I've got health conditions as well… why do they have to interrogate people?

Dave described not being believed about his health condition, interpreted as the assessor stigmatising through questioning the legitimacy of his benefit claim:

She said, “can you move it?”. I said “yeah, I can move it”. “Well it's not sore is it? So you've lied about that and everything”.

This reported speech suggests Dave experienced the conversation as invalidating and accusatory, even if the assessor was not as leading as this excerpt implies.

Miss Moneypenny appeared to justify the scrutiny and judgement of the assessor, suggesting internalised negative stereotypes linked to benefits:

They gotta ask those questions and make sure that the person is not doing wrong and pretending to be disabled.

There seems to be a strong moral dimension to Miss Moneypenny's defence of the assessment process, linked here to the possibility of benefit fraud.

#### 3.2.3 Disempowerment: ‘You've Got to Do It Though’

Analysis suggested all participants experienced powerlessness, tied to the risk of losing welfare:

You've got to do it though, it's important or they'll cancel your benefits. (AJ)

AJ demonstrates her understanding of the serious consequences, despite the explicit sense of coercion, with ‘cancel’ perhaps reflecting the assessor's use of language.

Passivity was interpreted as powerlessness:

They sort of sort it out for me they sort all me money out. (Miss Moneypenny)

Louise's personalised attributions framed the assessor, as the decision‐maker, as powerful within the assessment:

Then might give you money, might not give you money. We have to do whatever they say.

AJ appeared to feel forced to answer personal questions during the assessment, suggesting a sense of powerlessness:

It's quite scary and worrying… I didn't wanna answer them.

Despite noticing her own feelings about assessment, AJ expresses that she has had no choice in participating.

### 3.3 Relationship With the Assessor: ‘His Attitude Fucking Stunk’

All participants discussed how the fear and marginalisation of the assessment process impacted their relationship with the assessor, who often seemed to embody the system as expressed through their perceived humiliating and dismissive attitudes.

#### 3.3.1 Humiliation and Dismissal. ‘I Felt Humiliated in Front of Her’

Analysis suggested all participants experienced the assessor as humiliating and felt socially inferior during interactions (‘He spoke to me like shit’ Sue; ‘I thought she was a bit of a snob’ Miss Moneypenny). Miss Moneypenny clearly stated she felt humiliated and explained how this negatively affected her mood:

I felt humiliated in front of her… I got a bit down in the dumps over it really cus to be asked those kinds of questions, it's not nice.

Assessors experienced as impersonal seemed dismissive of potential emotional impacts of the assessment:

They'll just go through the questions and they'll be like can you do this, can you do that… they're that busy asking the questions they're not seeing how like you're reacting. (Natasha)

Louise described her assessor persisting when she became distressed and requested a break, interpreted as minimising emotional experience (‘the lady who's interviewing me she was like no we're just best to carry on’).

Only Jonathan described a helpful interaction with an assessor, ‘trying to be like, act like my buddy’ and help his understanding (‘dumb the questions down for me’). However, Jonathan's language was interpreted to suggest a patronising, rather than reasonable, adjustment, possibly reflecting the assessor's perceived ableism. The word ‘dumb’ has historically been used as an offensive and disablist term, and although rehabilitated somewhat through the use of ‘dumbing down’ in relation to simplifying screen‐play, still retains a very negative connotation. Jonathan's perception of ‘dumbing down’ as helpful may possibly, in turn, imply internalised stigma or that for him this phrase has lost its stigmatising association.

#### 3.3.2 Not Understanding Me: ‘They Don't Understand Me’

Five participants felt either misunderstood personally by assessors (‘They don't understand me’ Louise) or that ‘They don't really understand people with learning disabilities’ (Natasha). Natasha's construction appears to reset the power relationship through drawing attention to inadequacies of the assessor, rather than the assessed.

Analysis suggested that assessors also ignored what it meant to have an intellectual disability:

But I can't remember them asking me how I actually deal with my disability. (Bob)

Here, participant's accounts may suggest that the lack of assessor's authentic engagement, going through the motions perhaps, was another form of dismissive relating.

### 3.4 Others as a Safe Base: ‘Someone There That You Know, and You Trust’

All participants discussed the supportive role of others in relation to the benefit process. This was expressed in a sub‐theme related to different types of support and a sub‐theme highlighting different levels of independence.

#### 3.4.1 Support From Others: ‘I Just Love What They Done for Me’

All participants discussed helpful support from the 3rd sector (‘[name of charity] was supporting me’ AJ) and family (‘Mum and my auntie… was always there to help me’ Jonathan). Multifaceted support included filling in forms (‘She do'ed the sign… you know, tick the things’ Dave), helping with understanding (‘Some of the staff… are brilliant, cos they will help me read the letters off the Job Centre’ Louise), emotional support (‘I had my family and friends trying to calm me down and not panic as much’ Bob) and financial support (‘I got some money off my family’ Dave).

#### 3.4.2 Personal In(ter)dependence Payment: ‘When I'm on My Own I Panic’

Analysis suggested independence, dependence and interdependence were important to the experience of six participants. Scary and marginalising experiences appeared to increase dependency on others to navigate the system safely:

Don't like interviews anymore… I have to take either someone from [Name of charity] or another person with me. (Louise)

There was also the risk of financial dependency when benefits are stopped:

If next year I get my PIP stopped, I've got to live on [spouse's] money. (Sue)

Dave highlighted how support increased his confidence to do things independently, rather than promote dependence:

When I've got people around me. I'm ok. If I'm all on my own or something like that, or on the phone, I have to get someone else to erm speak to them.

Support appeared to improve participant's experiences (‘You're gonna be more relaxed at, erm, you're gonna be more like confident with yourself with someone there that you know and you trust’ Louise). In contrast, the negative impact of being alone was discussed by Jonathan:

Because when I'm on my own I panic, feel like I'm, my chest is about to pop out.

Sue appeared to seek reassurance and support from her spouse and carer who was present at her interview (‘I get my PIP now don't I baby?’) which initially suggested dependency. During the interview he fell, and Sue responded by care‐giving (‘Are you alright? You ok? You cut yourself… You ok, you alright?’ Sue). This was interpreted as interdependency, highlighting a mutual and reciprocal relationship. Louise described how she volunteers, also suggesting a reciprocal, supportive relationship, as opposed to what might be the expected support dynamic within this relationship:

I always help [name of charity] out a lot… I can't say no to them. (Louise)

Louise's description of her ongoing value to an external organisation conveys the warmth and affection of a close relationship, which empowers her contribution.

### 3.5 Responding With Empowerment: ‘That's Where I Really Shined’

Seven accounts suggest participants coped psychologically with the assessment process through resisting their disempowered positioning, either through challenging the assessor in the assessment interaction or sharing how they found self‐worth elsewhere.

#### 3.5.1 Resistance: ‘You're Not Getting Away With It’

Four participants described resisting the powerlessness they had felt in the assessment:

I said you do not speak to me like you're speaking to me, you're not getting away with it. (Sue)

I was like, yeah, if you [the assessor] ask the questions that I don't understand, then she'll have to answer them… on my behalf…I said well no, I'm not, I'm not, not talking to you anymore. (Louise)

Both Sue and Louise quote their own very direct, agentic and repeated speech to explain how they had resisted perceived disrespect and tried to regain control from their excluded positioning.

Natasha suggested she leave ‘feedback’ on staff performance to hold the system accountable: ‘I think they should send you a letter the week after with like smiley faces on or sad faces on and say what do you give this appointment.’ This suggests she was trying to re‐empower herself through adopting a consumer positioning through which *she* reviews the assessor.

#### 3.5.2 Finding Value: ‘Confidence and Belief in Myself’

Demonstrating that they hold productive social and moral value outside of this threatening, alienating and overwhelming process was important for four participants in contrasting their experiences of welfare assessment. Jonathan discussed the things he valued about employment: ‘I think, I think I enjoyed the erm, going around [local area], seeing all the new places, making new friends’. Louise described feeling valued in her volunteer role, about which she felt ‘very good… pleased with myself’. Jonathan discussed how specialist college attendance helped him to value himself (‘That's where I really shined’) and said his experience of employment, volunteering, and college ‘brought me confidence and belief in myself.’

Two participants spoke with pride about their achievements (‘I've got loads [of certificates]. Two files, one in grey and one in maroon… not only that I've got good attendance certificates’ Sue) as well as their intrinsic personal attributes (‘My, my kindness. And [Name] can vouch for that’ Louise). Here Louise appears to reject the criteria of the assessment, seeking to define how she feels her value be assessed. Sue is clear about her moral code which appeared to help her assert her equal value to the assessor: ‘…if they treat me with respect, I'll treat them with respect.’

Notably, participants tended to use much more active language throughout this theme, often appealing to external sources (consumer‐style reviews of service received, supporters or certificates) to regain power and control.

## 4 Discussion

This research explored the experience of people with intellectual disabilities, who have been subject to welfare assessments related to their benefit eligibility. Five GETs were generated through analysis of the participant's narratives: (1) *Living in fear*; (2) *the system is marginalising*; (3) *Relationship with assessor*; (4) *Others as a safe base* and (5) *Responding with empowerment*.

The themes *living in fear* and *marginalisation* support literature suggesting eligibility assessments in the UK welfare system are experienced by claimants as largely negative (Headway 2018; Webb and Albert 2022; Machin and McCormack 2023), difficult to understand (Allen et al. 2016) with claimants not feeling listened to (Gray 2014, 2017; United Nations 2016) and procedural efficiency prioritised over rapport and developing trust (Porter, Pearson, and Watson 2021). The analysis also supported Litchfield's (2014) suggestion that some people with intellectual disabilities may find it difficult to fully express their needs in a functional assessment, illustrated by participants who felt they had lost their benefits due to ‘performing well’ and focusing on their strengths.

The analysis provides empirical evidence expanding on the findings of Moth and Lavalette (2017) and Pybus et al. (2021) samples of people with mental health difficulties subject to functional assessments, to the current sample of those with intellectual disabilities. Mechanisms of fear, invalidation, double binds, and ‘shaming and blaming’ were also present in the current study within the themes *living in fear* and *marginalisation*. This suggests some similar experiences of welfare assessments across groups, however group and individual differences are evident. Participant experiences suggest UK functional benefits assessment practice lacks the ‘reasonable adjustment’ (Equality Act 2010) or ‘reasonable accommodation’ (UN Convention on the Rights of Persons with Disabilities 2006) to communication needed to ensure that people with intellectual disabilities enjoy human rights on an equal basis with others.

In the current analysis, the *reinforcing intellectual disability stigma* sub‐theme was present in more participant's accounts than the *reinforcing benefit stigma* sub‐theme, suggesting that the assessment process was more likely to reinforce stigma associated with having an intellectual disability. This supports Scior and Werner's (2016) argument that having an intellectual disability remains highly stigmatising, as this experience appeared to be more salient to the participants than benefit stigma. In this sample, the generally negative experience of undergoing welfare assessments documented in the literature not only applied to participants, but was compounded by their experience of having an intellectual disability. In the context of literature that demonstrates people with intellectual disabilities feel stigmatised and powerless across multiple spheres of their lives (Scior and Werner 2016), the welfare assessment may be a microcosm of people's general experiences of having an intellectual disability.

Within this analysis, the theme of *marginalisation* illustrates how welfare assessment processes reinforce stigma, disempowering those with intellectual disabilities, serving to socially marginalise them. *Disempowerment* was demonstrated across all participants, resonating with literature suggesting UK welfare claimants experience feelings of powerlessness (De Wolfe 2012; Ploetner et al. 2020). The *reinforcing stigma* sub‐theme suggested some participants internalised stigma while some did not, supporting the literature suggesting not all stigma is internalised (Corrigan and Watson 2002).

Discussion of *others as a safe base* was more frequent than *responding with empowerment*. This suggests that support from others could promote independence, reflecting critical disability literature that challenges the traditional ideology of independence (e.g., Morris 2004). Interdependency was demonstrated in the *Personal In(ter)dependence Payment* theme; this reflects current thinking around independence that highlights the importance of building mutual relationships between people with intellectual disabilities, their carers, and services (Giri et al. 2021).

Some participants expressed *finding value* in employment and education settings. They discussed having opportunities to travel, make new friends, and feel equal to peers during employment. This reflects research that suggests meaningful roles can reduce the emotional consequences of stigma (Dagnan and Sandhu 1999). However, for meaningful employment to be an option for people with intellectual disabilities, it is argued that the welfare system would need to address their restricted employment opportunities and offer support (Redley 2009). Furthermore, other roles may be just as beneficial, as illustrated in the *Personal In(ter)dependency* sub‐theme.

### 4.1 Reflections

The research team consisted of like‐minded academics, who shared political views and practice elements of social justice activism. This created a left‐aligned activist perspective permeating the research. While the team focused on the harm caused by the welfare assessment, another analysis may have interpreted data differently, highlighting the value of providing some sort of state assistance to people with intellectual disabilities.

Research supervision highlighted that occasionally direct questions about negative experiences were avoided by the interviewer to ‘protect’ the participants from emotional distress. This could be viewed as an enactment of the researcher's implicit assumptions associated with their own privilege and power; that people with intellectual disabilities need support and protection. The impact of this bias may have disempowered some participants by reducing space to share negative experiences and limiting the data.

### 4.2 Limitations

The findings are based upon the experiences of people with intellectual disabilities who were recruited via two charities, promoting equality and social inclusion for people with learning disabilities. While this probably contributed to homogeneity within the sample, it may have influenced how the participants, who had above average experience of employment, and were likely exposed to social models of disability and advocacy‐based support, made sense of their experiences. Future research might consider the potentially very different experiences of other people with intellectual disabilities. The choice of IPA as a method, whilst an accepted method for research with people with intellectual disabilities (Rose et al. 2019) may also have excluded some people from the study.

Intellectual disability diagnosis was assumed through service eligibility criteria. Therefore there is a small chance that some participants may not have met formal diagnostic criteria. However, the subjective experience and self‐disclosure of having an intellectual disability was congruent with IPA principles and the researcher's epistemological stance.

Interviews were mostly conducted in the presence of a staff member to safeguard participant wellbeing. This may have impacted the emphasis on the third sector as a supportive and safe base, due to potential desire to please and unwillingness to criticise support from staff. While people with intellectual disabilities were consulted throughout the research process, having a member of the research team who has an intellectual disability may have strengthened the analysis by broadening interpretations made about the data.

The research was conducted during the global COVID‐19 pandemic. Interviews were virtual, which increased recruitment bias as only people with online access were included. The participants and the interviewer were living in a period of uncertainty, fear and anxiety. This may have impacted the way the interviews were conducted, the things that felt important to the participants and the researcher's interpretations. Many people were not able to contact their support network during this time, which may have impacted the research by highlighting the importance of social connections. Furthermore, the participants had reduced contact with the DWP within this timeframe as all assessments were suspended.

### 4.3 Multi‐Level Interventions: Practice Implications

**FIGURE 1:**
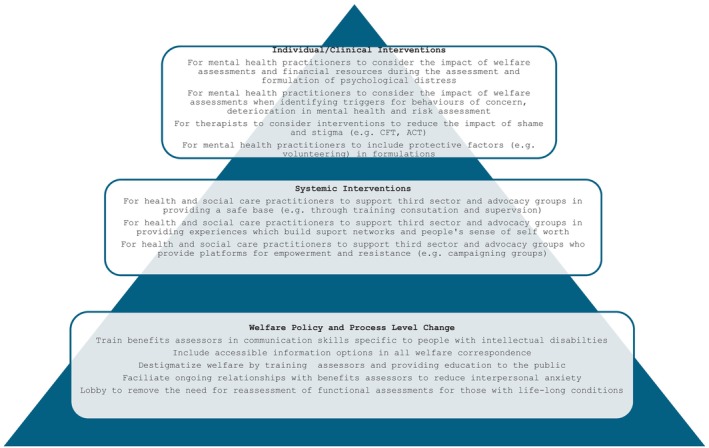
Multi‐level interventions for practitioners to support and improve the experience of people with intellectual disabilities undergoing welfare assessments.

Figure 1 summarises recommended interventions based on the analysis. Individual‐level therapeutic interventions which focus on reducing shame, such as Compassion Focussed Therapy (CFT; Gilbert 2010) and Acceptance and Commitment Therapy (ACT; Hayes, Strosahl, and Wilson 2012) could be employed by therapists and psychologists working in clinical services for people with intellectual disabilities, to understand and target the distress and stigma experienced in relation to welfare assessments. Health and Social Care Practitioners could contextualise people's emotional distress by considering the impact of PIP assessments, rather than individualising and pathologizing their presenting difficulties.

At a systemic level, Health and Social Care practitioners could support third sector and advocacy groups, who provide a safe base for people with intellectual disabilities undergoing welfare assessments and facilitate empowerment and self‐advocacy. Attempts to build on the sense of accomplishment participants reported feeling about their voluntary and paid employment would likely create a more productive and meaningful scaffolding towards higher levels of paid employment where this is desired.

Health and Social Care practitioners could also engage in wider activities to reduce economic distress, such as using their social status and power to campaign for change within welfare policy and processes, as outlined in Figure 1. They could include lobbying for psychological thinking within the system. These policy level changes would serve to reduce the fear of losing benefits, improve the relationship between assessors and those being assessed, reduce the confusion over letters described by study participants through making information more accessible, foster a sense of achievement and facilitate a person‐centred approach. Recent research by Cantrell, Weatherhead, and Higson (2021) explored clinical psychologist's experiences of working in the context of the benefit system. They found that participants believed they could and should influence higher‐level change in the welfare system, however emphasis was placed on the need for professional support and leadership to engage in this work. This could focus on more choice and flexibility in the ways that people are assessed and a shift towards an interactional and interpersonal style of correspondence to humanise the experience of assessments (Porter, Pearson, and Watson 2021; Webb and Albert 2022).

### 4.4 Future Research

The fear created by welfare assessments suggested by this exploratory research could be investigated within a larger and more diverse sample. Future research may consider how experiences of the benefits system might reinforce intellectual disability stigma. Only some participants showed evidence of internalised stigma and it would be useful to explore protective factors, as well as exploring the effectiveness of the practical interventions proposed to reduce the psychological harms of welfare assessment, suggested within the narratives of this small sample.

## 5 Conclusion

People with an intellectual disability subject to functional assessments for UK welfare benefits experience the system as scary and oppressive. The process resonated with past negative experiences of assessments and reinforced the stigma and marginalisation associated with having an intellectual disability and, to a lesser extent, claiming benefits. Participant interactions with both the system and the assessor maintained a power imbalance. Participants responded with empowerment, however their relationships with safe and supportive others were the most beneficial in improving their experience. From this analysis, systemic approaches to improving the benefit assessment process for people with learning disabilities seem more likely to be helpful than individual interventions to cope with disempowerment or stigma. This might include making changes to the claiming process and benefit system and the facilitation of meaningful relationships within and beyond the benefit system.

## Ethics Statement

Ethical approval was granted by the University of Liverpool's Central Ethics Committee on the 28th January 2021.

## Consent

An accessible participant information sheet and consent form were developed based on guidance from the Department of Health and National Research Ethics Service (DH, 2010; NRES, 2011) and consultation with people with intellectual disabilities. During a pre‐meeting, the PIS was shared via Zoom's screen‐share function and read through by the researcher, who answered any further questions. Six participants agreed to participate during the pre‐meeting, while two contacted Mencap staff the following week to confirm participation. Consent was audio‐recorded following review of the consent form at the beginning of each interview.

## Conflicts of Interest

The authors declare no conflicts of interest.

## Data Availability

Data from this study are not publicly available as participants did not consent to this.
